# Ferroptosis-related *NFE2L2* and *NOX4* Genes are Potential Risk Prognostic Biomarkers and Correlated with Immunogenic Features in Glioma

**DOI:** 10.1007/s12013-022-01124-x

**Published:** 2023-01-11

**Authors:** Li Lin, Xiaona Li, Shunda Zhu, Qingshan Long, Yongzhen Hu, Liyang Zhang, Zexin Liu, Bo Li, Xuesong Li

**Affiliations:** 1 Huizhou Third people’s hospital, Guangzhou medical university, Huizhou, 516002 Guangdong P.R. China; 2 Department of Radiology, the Second Affiliated Hospital, School of Medicine, South China University of Technology, 516002 Guangdong, P.R. China

**Keywords:** Ferroptosis, Glioma, Immune infiltration, Prognostic biomarker

## Abstract

Ferroptosis is a newfound mode of regulated cell death that may have potential to associate with prognostic or diagnostic factors in glioma. In this research, 5 genes related to glioma were screened through the FerrDb database, and we analyzed the combination between genes and glioma of survival and prognosis via TCGA, GEPIA, TIMER, and other databases. Survival curve and prognostic analysis showed that the overexpression of *NFE2L2* and *NOX4*, respectively, has a remarkable link with a worse prognosis in glioma. Then, the association between the expression of the two genes and tumor-infiltrating immune cells level was explored based on the GSCA, and the immunity of *NFE2L2* and *NOX4* based on the TISIDB database was also investigated. In glioma, especially GBM, there is a strong association between gene expression and immune infiltration, even in macrophages, nTreg, and Th2 cells, which play immunosuppressive functions in TME. In conclusion, these results indicate that *NFE2L2* and *NOX4* could be risk prognosis biomarkers in glioma, and they bound up with immune infiltration and tumor immunity in tumorigenesis.

## Introduction

Glioma is a tumor caused by lesions on glial cell that is an important part of the central nervous system, which accounted for 1.6% of all new tumor cases and 2.5% of all cancer-related deaths in 2018 [1, 2]. According to the malignancy degree, gliomas are categorized into four histological grades I-IV by WHO [3]. Glioblastoma (GBM) is a type of histological grade IV glioma with only 5% of survival rate within 5 years [4]. At the present, surgical resection, chemotherapy, postoperative radiotherapy, and immunotherapy would be considered to glioma clinical treatment [5–7]. Nevertheless, the median survival rate of GBM patients is no more than 15 months yet [8]. Moreover, LGG also has little response to the standardized therapeutic method. Those issues, especially therapeutic resistance and tumor recurrence, lead to treatment failure and poor prognosis [9, 10]. In current clinical practice, repeatedly mutated genes such as *IDH1*, *IDH2*, *TP53*, *EGFR*, and *ATRX* are identified as prognostic factors in LGG [11–13]. Meanwhile, other molecular markers, including 1p/19q co-deletion and *MGMT* promoter methylation, are also considerable prognostic factors in LGG [14]. Whereas these clinicopathological and genetic factors can not accurately assess survival outcomes. Thereby, an urgent requirement for the underlying mechanism of glioma development needs to be solved, and to gain insight into the effective factors influenced the diagnosis and prognosis of glioma patients to locate new diagnostic biomarkers and find potential therapeutic targets.

Cell death is a physiological mechanism of body to maintain its normal functions through necrosis and programmed cell death (autophagy and apoptosis) [15]. The connection between cell death and disease has been illustrated after the discovery of different types of cell death such as apoptosis [16], ferroptosis [17], and pyroptosis [18]. Some reported that cell death plays a significant role in the development of gliomas, and it also affects pathological classification and sensitivity to radio-chemotherapy [19, 20].

Ferroptosis is a programmed oxidative cell death that is characterized by highly iron-dependent lipid peroxidation [17]. In recent years, ferroptosis has received widespread attention. An increasing number of pertinence between ferroptosis and neurological diseases such as cancer, stroke, and ischemia-reperfusion injury have been found [21, 22]. Moreover, recent studies have revealed that ferroptosis is a promising anticancer strategy for overcoming therapeutic resistance [23–25] and can be applied to a lot of anticancer regimens, and there are some of these regimens have been approved by U.S. Food and Drug Administration [26, 27]. While the pathway of diagnostic factors in ferroptosis has not been clarified yet, and there are few studies focus on ferroptosis of glioma in molecular levels. Thereby, it is valuable to lucubrate the association of ferroptosis-related genes in glioma and the prognostic value behind it.

In addition, some further studies show that cancer progression and recurrence are not only affected by the tumor’s underlying genetic changes, but also by the tumor microenvironment (TME) [28–30]. TME is very complex, including cellular components such as cancer cells, immune cells, endothelial cells and noncellular components such as cytokines and chemokines [31, 32]. Increasingly data sustained that immune cells in TME take a significant role in tumor progression and recurrence [33–36]. The dynamic changes of TME reflect the evolutionary nature of tumors, involving tumor immune escape, tumor growth, and metastasis. Therefore, a deeper understanding of TME and its related mechanisms are also crucial to the search of tumor prognostic factors.

In this study, we aimed to delve into the prognostic implications of ferroptosis-related genes and immune infiltrates in glioma. Herein, we screened 5 ferroptosis-associated genes which were related to glioma through FerrDb database, and then systematically analyzed the correlation between gene expression with prognosis and immune infiltration of glioma through database systems such as TCGA, GEPIA and TISIDB. Our findings suggested that the ferroptosis-associated genes *NFE2L2* and *NOX4* are potential risk prognostic biomarkers and are correlated with immune infiltration in glioma.

## Methods

### Identification of ferroptosis-associated genes related to glioma and data collection

FerrDb is a database for preserving and providing information about ferroptosis-related markers and regulators, it also can be used to identify ferroptosis-associated diseases. We identified five ferroptosis-associated genes (Table 1) that have been tested in human glioma with different research methods in FerrDb (http://www.datjar.com:40013/bt2104/). And the ferroptosis-related signaling pathway that the five genes involved in was also analyzed.

**Table 1:** A list of ferroptosis-associated genes related to glioma, was screened through the FerrDb database

Symbol	Name	Test in	Test method
KEAP1	Kelch like ECH associated protein1	Human, rat	Cell viability analysis
*NFE2L2*	Nuclear factor, erythroid 2 like 2	Human, rat	Cell growth assay
*NOX4*	NADPH oxidase 4	Human, rat	Western blot, LDH release assay, siRNA, cell death
*ATF4*	Activating transcription factor 4	Human	siRNA, cell transfection, cell viability, RT-PCR, Immunoblotting
*ASCL4*	Acyl-CoA synthetase long chain family member 4	Human	Western blotting, 5-HETE, LDH immuno-fluorescence, CCK8

The obtained five genes’ expression data in tumor and normal tissue samples corresponding to 33 cancer types were analyzed from TCGA (https://www.cancer.gov/about-nci/organization/ccg/research/structural-genomics/tcga). And the immune histochemical images of protein expression in glioma and normal tissues were obtained from Human Protein Atlas (HPA) (https://www.proteinatlas.org/).

### Survival and prognosis analysis

The relationship between gene expression and patient’s survival (OS: overall survival; DFS: disease-free survival) in GBM and LGG were obtained from GEPIA(http://gepia.cancer-pku.cn/) and TIMER (https://cistrome.shinyapps.io/timer/). Then, the relationship between gene expression and glioma histology was analyzed from the TCGA database. The prognostic ability was evaluated by ROC and AUC analysis using the package of “survival ROC” in R. We also conducted multivariate Cox proportional hazards model analysis to evaluate whether a gene can be used as an independent prognostic factor in GBM and LGG using the Timer database. The hazard ratio (HR) and 95% confidence intervals were calculated via univariate survival analysis.

### Correlations between gene expression and immunogenic features in glioma

Gene Set Cancer Analysis (GSCA, http://bioinfo.life.hust.edu.cn/GSCA/#/) is an integrated platform for genomic, pharmacogenomic, and immunogenomic gene set cancer analysis. The immune infiltration levels were quantified using GSCA, which provided immunogenomic analysis was performed by the ImmuCellAI algorithm on 24 immune cells. We explored the correlation analysis between gene expression and immune infiltration levels in glioma (GBM and LGG). The heatmap and spearman correlation analysis between gene expression and immunity in glioma (GBM and LGG) were analyzed from TISIDB (http://cis.hku.hk/TISIDB/).

### Statistical analysis

Performing statistical analyses with R software and SPSS software. By long-rank test, the correlation between critical factors and overall survival rate of patients had been analyzed that based on the survival and survminer R package. Spearman was applied to identify the relevance of genes and immune infiltration. Data were considered significant at *p* < 0.05.

## Results

### Identification of 5 ferroptosis-associated genes

We screened ferroptosis-associated genes related to glioma through the FerrDb database. As shown in Table 1, there were 5 ferroptosis-associated genes (*KEAP1*, *NFE2L2*, *NOX4*, *ATF4*, *ASCL4*) that have been tested in human glioma with different research methods. As shown in Fig. S1, 5 genes play an important role in the process of ferroptosis through different signaling pathways. Kelch like ECH associated protein1 (KEAP1) was defined as a significant factor that regulates the antioxidant response pathway and restrains erythroid-1 like 2 (*NFE2L2*/*NRF2*) which is a transcription factor [37]. While, *NFE2L2* is the key factor that manages lots of cell protection genes in transcription, which genes involved in iron metabolism, oxidative defense, and redox signaling during ferroptosis [38]. And NADPH oxidase (NOX) is a family of enzymes that produce ROS. Studies have shown that *NOX4*, as the main isotype in astrocytes, can promote the ferroptosis of astrocytes through lipid peroxidation induced by oxidative stress [39, 40]. Acyl-CoA synthetase long-chain family member 4 (*ACSL4*), catalyze fatty acid metabolism, has the potential to be a biomarker of specificity and promoter of ferroptosis since the ACSL4 overexpression can increase PUFA content in phospholipids [41–44]. Activating transcription factor 4 (*ATF4*) is an essential link between ER stress and oxidative damage, contributing to ferroptosis in a tumor-type-dependent manner mediated by a signal pathway such as ATF4–HSPA5–GPX4 [45, 46].

### Prognostic significance of ferroptosis-associated genes in glioma

**Fig. 1:**
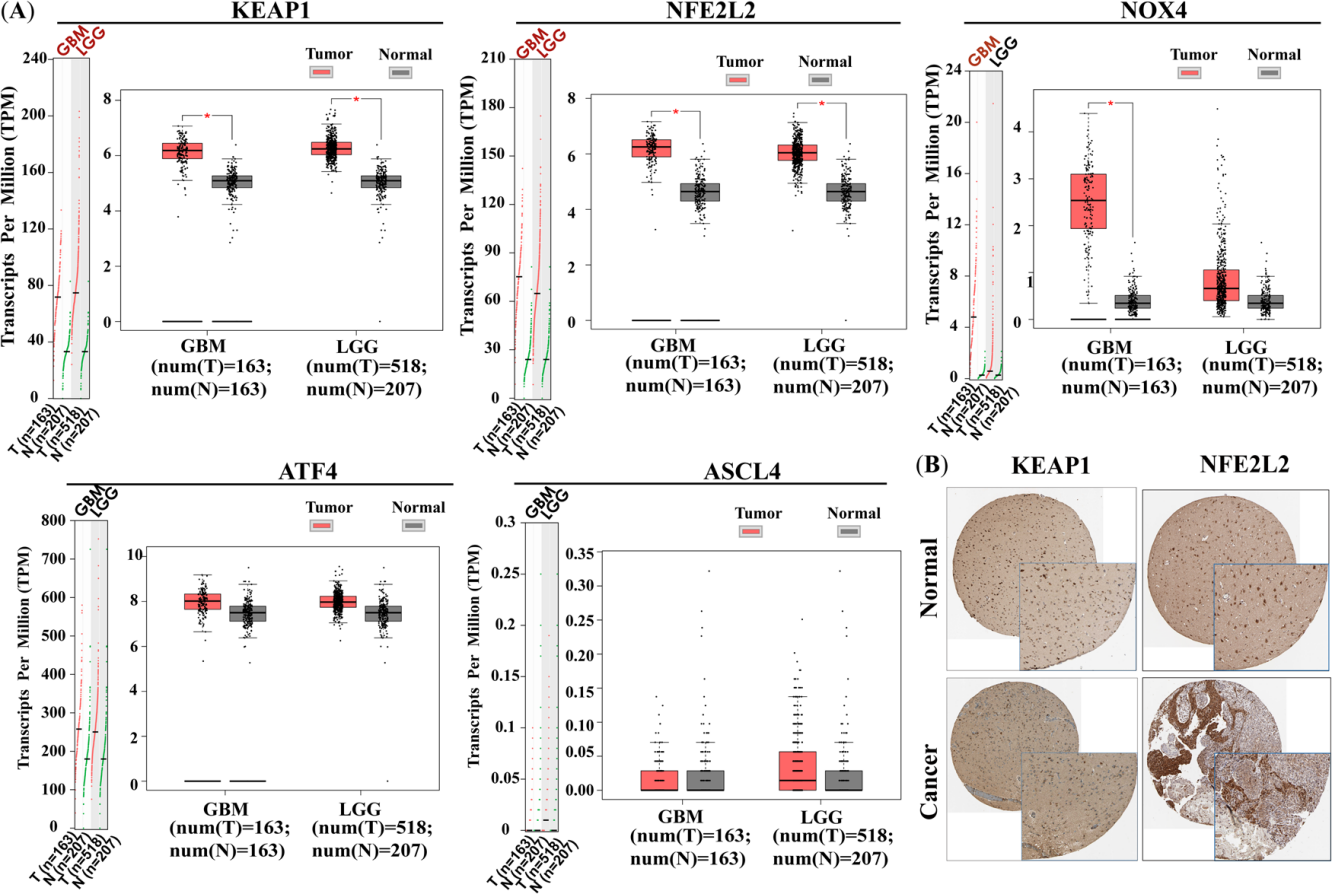
mRNA and protein expression of ferroptosis-associated genes in glioma and normal tissues. **A** Profile and box plots show gene mRNA expression in GBM/LGG (red) and normal tissue samples, *p < 0.05; **B** Immuno-histochemical images from HPA database show *KEAP1* and *NFE2L2* protein expression in glioma and normal tissues

To investigate if ferroptosis-related genes are associated with the progression of glioma, we firstly obtained the gene expression data in human pancancer from the GEPIA database. As shown in Fig. S2, the expression of *KEAP1*, *NFE2L2*, and *NOX4* were generally higher than normal level of body in more than 20 tumor tissue samples. However, *ATF4* showed reversed expression trends with low expression in most tumor tissues compared to normal tissues. As for the expression in glioma, the results are shown in Fig. 1, *KEAP1* and *NFE2L2* showed a near 2~3 fold increase in both GBM and LGG, and the *NOX4* showed a nearly 20-fold increase in GBM. While the expression of *ATF4* and *ASCL4* did not be significantly different in GBM and LGG compared to normal tissues. Then, the Human Protein Atlas (HPA) database analysis (Fig. 1B) also shows that *KEAP1* and *NFE2L2* expression is significantly higher in glioma tissues compared to normal tissues.

**Fig. 2:**
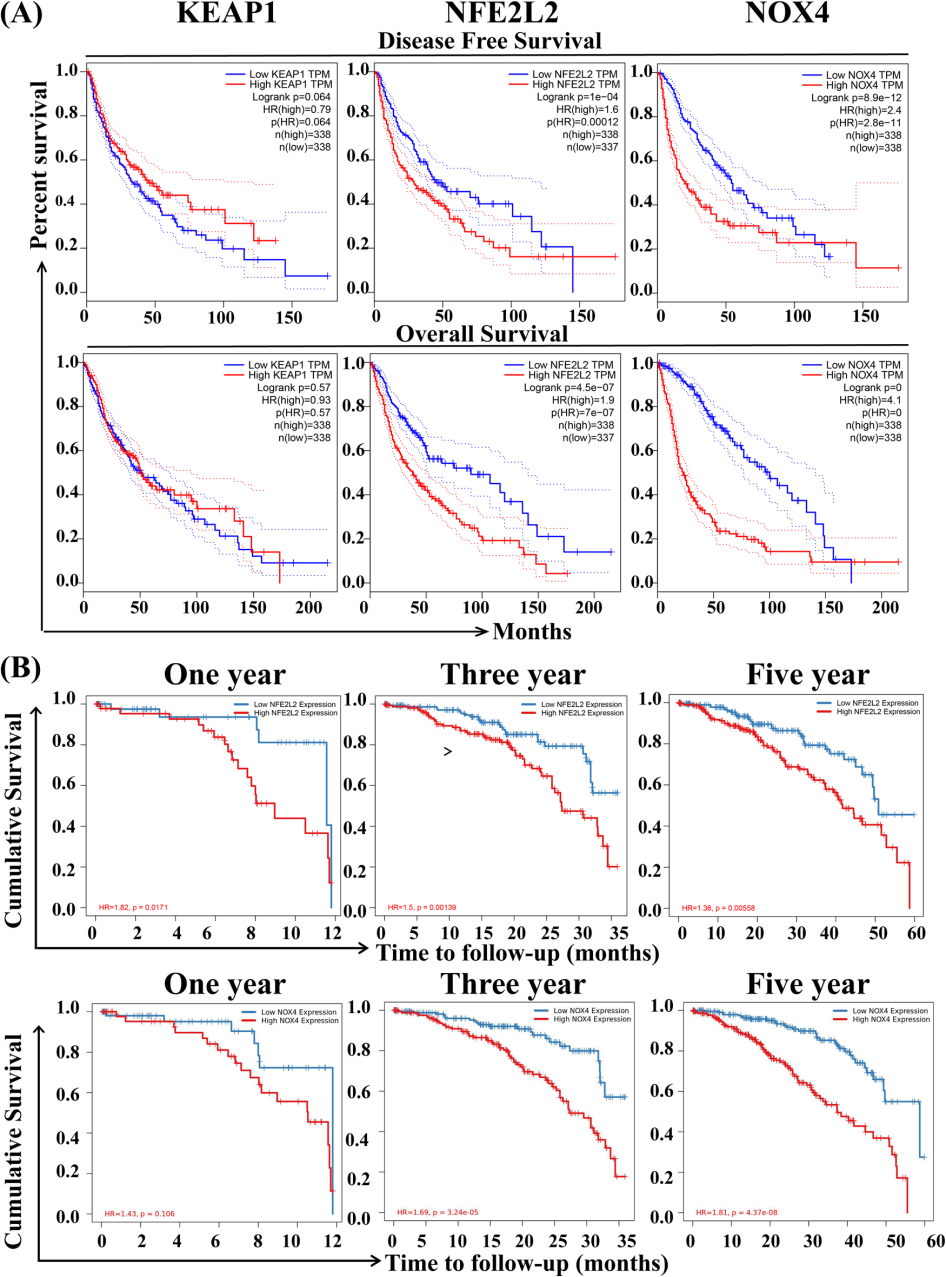
Relationship of ferroptosis-related gene expression with patients’ survival in GBM and LGG. **A** Disease free survival and overall survival curves of *KEAP1*, *NFE2L2*, and *NOX4* in glioma patients were analyzed by GEPIA. **B** 1, 3, 5-year cumulative survival rate of glioma patients correlated with *NFE2L2* and *NOX4* expression analyzed from TIMER database

The ferroptosis-related genes expression analysis showed the relationship of *KEAP1, NFE2L2*, and *NOX4* expression with patients’ survival in GBM and LGG, and disease-free survival (DFS) and overall survival (OS) were performed by GEPIA (Fig. 2A). Notably, the DFS and OS were significantly shorter in patients with high expression of *NFE2L2* and *NOX4* than in those with low expression of *NFE2L2* and *NOX4*. However, *KEAP1* expression was ineffective association with the survival curve. This result implied that *NFE2L2* and *NOX4* could be the key product that caused poor prognosis in glioma patients. Furthermore, we analyzed the correlation of 1, 3, and 5-year cumulative survival rates of glioma patients with *NFE2L2* and *NOX4* expression. As shown in Fig. 2B, the cumulative survival of glioma patients with low *NFE2L2* or *NOX4* expression was significantly longer than that of glioma patients with high expression. And the results also showed that the HR value of the survival curve gradually increased with time lapse, which means that the mortality of glioma patients with high *NOX4* expression gradually increased with the prolongation of time. In conclusion, the eccentric expression and appearance of *NFE2L2* and *NOX4* are risk factors in glioma.

**Fig. 3:**
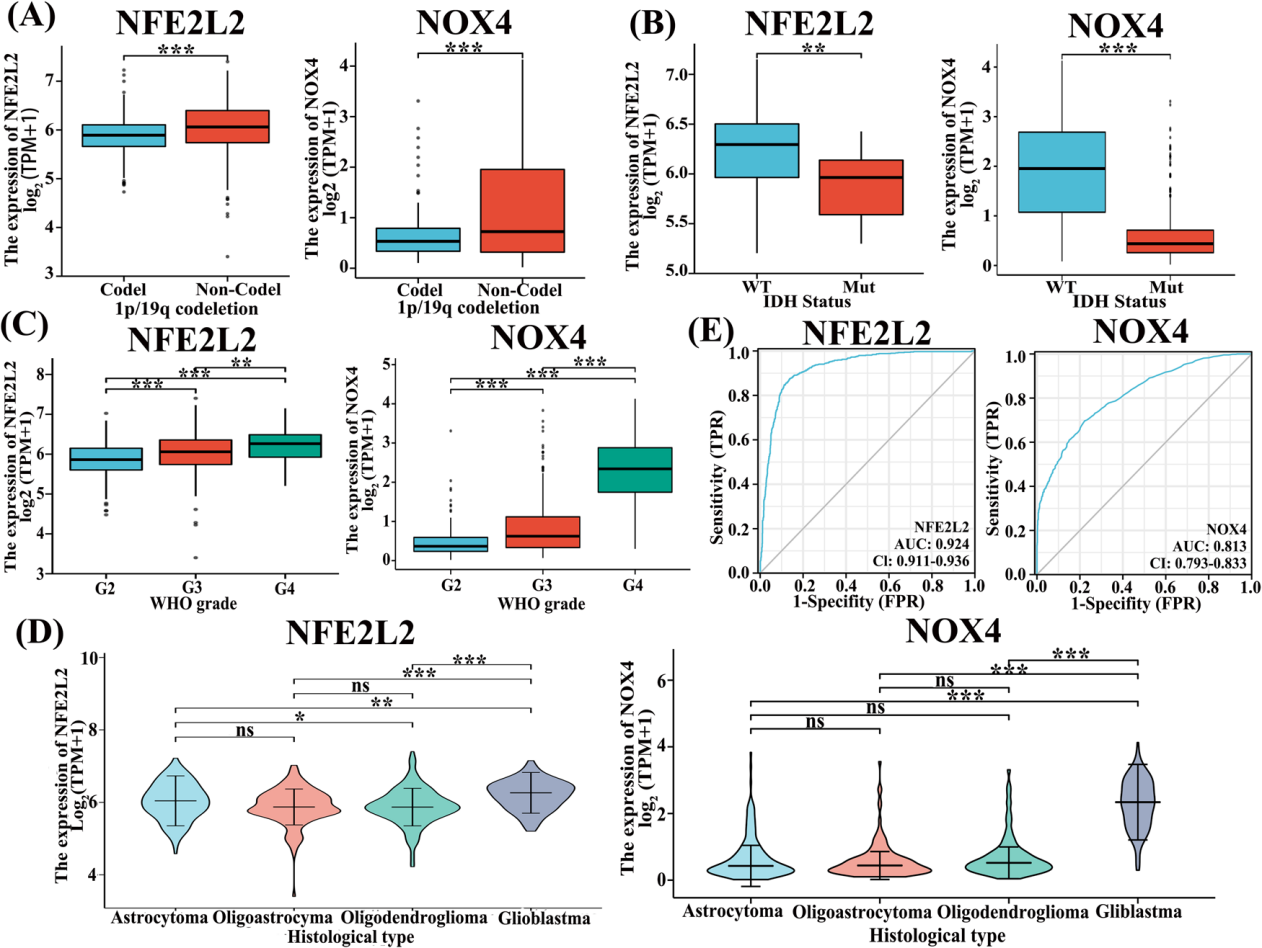
The association of gene expression (FPKM) with glioma histology and prognostic biomarkers in the TCGA database. **A**–**D** Correlation of *NFE2L2* and *NOX4* expression with (**A**) 1p/19q codeletion status, (**B**) IDH mutation status, (**C**) Grade and (**D**) Histology. *p < 0.05, **p < 0.01, ***p < 0.001. **E** ROC curve showed the predictive efficiency of gene expression

From the result of silico analysis by the TCGA dataset, it showed that IDH mutation and chromosome 1p/19q co-deletion are critical biomarkers that direct treatment and prognostication of glioma [47]. We found that low expression of *NFE2L2* and *NOX4* was associated with IDH mutated and 1p/19q codeletion group (Fig. 3A, B). Besides, the expression of *NFE2L2* and *NOX4* was positively correlated with the WHO grade of glioma (Fig. 3C). This means that the expression levels of *NFE2L2* and *NOX4* would increase with the glioma development. The analysis results (Fig. 3D) also showed that the two-gene expression was related to histology. As shown in Fig. 3D, the expression of *NFE2L2* increases with the histological severity of glioma, and *NOX4* is also significantly increased in the gradeIV glioblastoma. Besides, according to scores of pathological features, prognostic values were determined from the ROC curves by calculating the area under the curve (AUCs) of the gene expression. We observed that the prognostic value of those two genes in gliomas AUCs are 0.929 and 0.813, respectively (Fig. 3E).

**Fig. 4:**
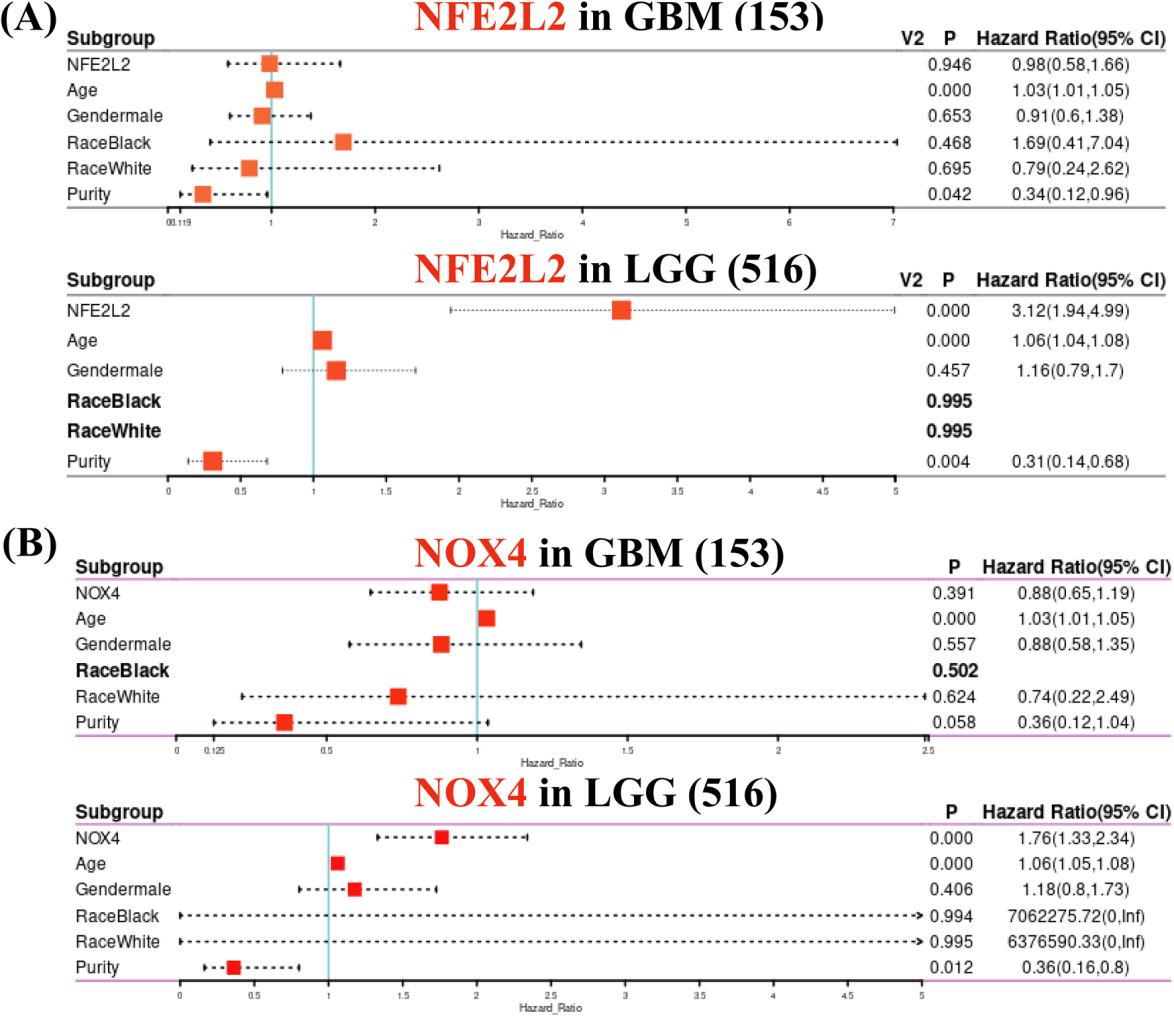
Cox proportional hazards model analyses of *NFE2L2* (**A**) and *NOX4* (**B**) mRNA levels and clinical factors predictive of overall survival in GBM and LGG

To further determine whether gene expression represented an independent prognostic index in glioma, Cox proportional hazard model analyses of *NFE2L2* and *NOX4* mRNA levels and clinical factors predictive of overall survival in GBM and LGG were performed using the Timer database. As shown in Fig. 4, there is only age that is considered as a risk factor (*P* < 0.001, Hazard Ratio, HR > 1) that significantly affected prognosis in GBM. However, in addition to age, *NFE2L2* and *NOX4* can significantly affect the prognosis in LGG. On the other side, the HR values of *NFE2L2* and *NOX4* were 3.12 and 1.76, respectively, indicating that these two genes are the risk factors that affect the prognosis of LGG. Thereby, the risk of death is in direct proportion to the expression of the two genes. The above results proved that *NFE2L2* and *NOX4* can be used as prognostic risk factors in gliomas, especially in low-grade gliomas.

**Fig. 5:**
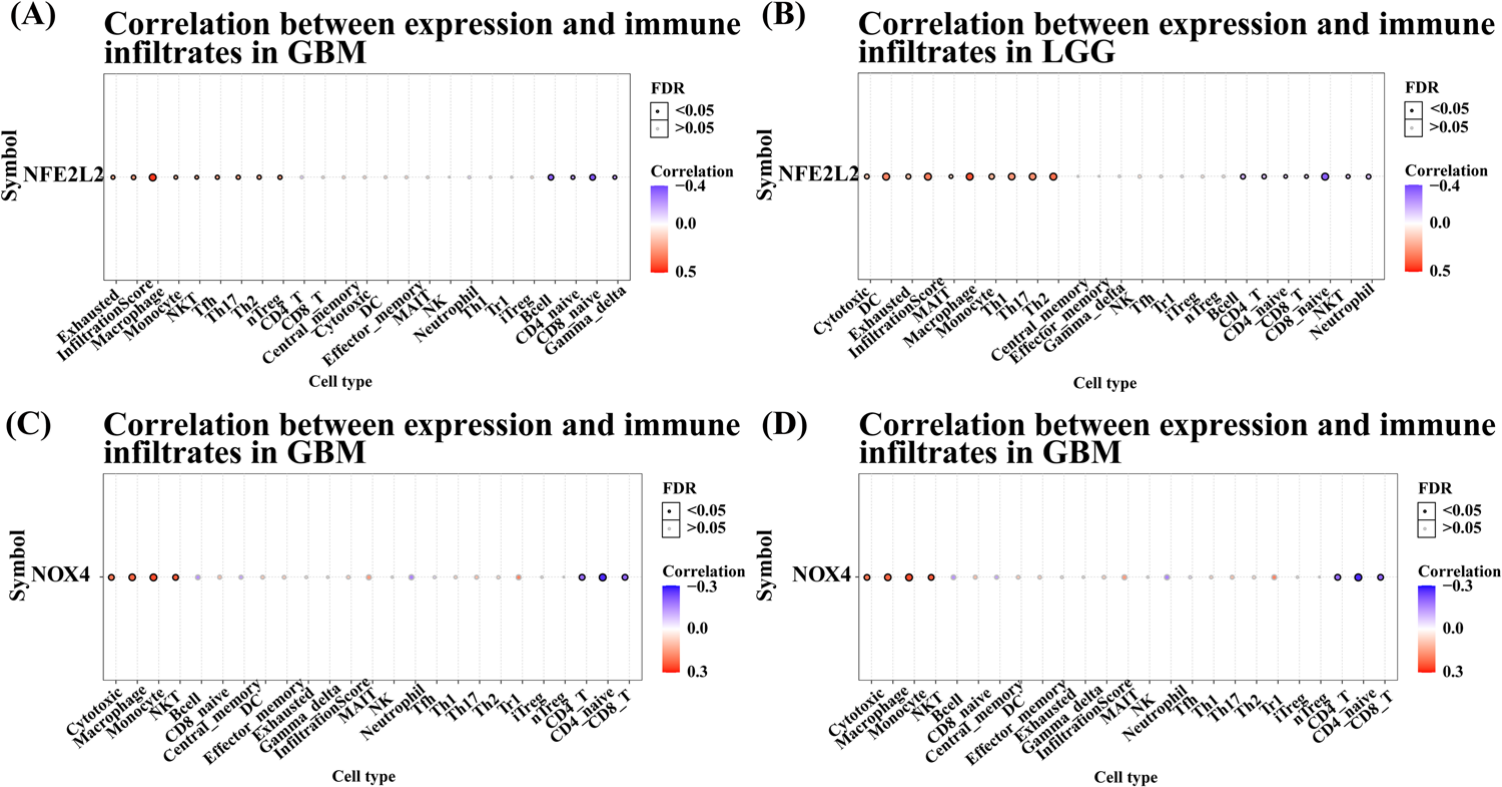
Correlation analysis between *NFE2L2* and *NOX4* expression and immune infiltrates in glioma (GBM and LGG). Correlation between *NFE2L2* expression and immune infiltrates in GBM (**A**) and LGG (**B**); Correlation between *NOX4* expression and immune infiltrates in GBM (**C**) and LGG (**D**)

The interaction of tumors and immune cells will facilitate the anticipation of immunotherapy response and the development of novel immunotherapy targets. The interaction between tumors and immune cells plays a crucial role in the occurrence, development, and treatment of cancer. Besides, tumor-infiltrating immune cell is an independent predictor of cancer status and patients’ OS [48]. The affiliation of gene expression and immune infiltration levels in glioma was probed by using GSCA in this study. Our results showed the correlation of *NFE2L2* expression level with high immune infiltration both in GBM and LGG, while *NOX4* expression mainly correlated with high immune infiltration in LGG (Fig. 5). These analyses also revealed that high expression levels of *NFE2L2* could significantly increase the immune infiltrating levels of exhausted T cells, macrophages, monocyte cells, NKT, Tfh, Th17, Th2, and nTreg cells, especially of macrophages (Cor. = 0.46) and nTreg cells (Cor. = 0.26), while passive tie was detected for levels of CD8 naïve T Cells (Cor. = −0.37), B cells (Cor. = −0.35), CD4 naïve T cells and Gamma delta T cells in GBM (Fig. 5A, Fig S3A). In LGG, the relationships between *NFE2L2* expression and immune infiltration levels were a little different (Fig. 5B, Fig. S3B). *NFE2L2* expression was positively associated with cytotoxic cells, DC, exhausted T cells, MAIT, macrophage (Cor. = 0.42), monocyte cells, Th1, Th17, and Th2 (Cor. = 0.36), while negatively associated with B cells, CD4 T cells, CD4 naïve, CD8 T cells, CD8 naïve cells, NKT (Cor. = −0.14) and neutrophil cells (Cor. = −0.16). Similarly, as shown in Fig. 5C and Fig. S4C, *NOX4* expression levels were positively associated with 4 types of infiltrating immune cells, including cytotoxic T cells, macrophages (Cor. = 0.23), monocyte cells (Cor. = 0.26) and NKT cells (Cor. = 0.24) and negatively associated with 3 types of infiltrating immune cells, including CD4 T cells, CD4 naïve T cells (Cor. = −0.27) and CD8 T cells in GBM. However, in LGG, high *NOX4* expression could meaningfully enhance the infiltrating levels of many kinds of T cells including CD8 T cells, cytotoxic T cells, DC, effector memory cells, gamma delta T cells, MAIT, Tfh, Th1, Th2, Tr1, and nTreg cells. Moreover, a negative correlation exists between the NOX4 expression level and infiltration levels of CD4 T cells and neutrophil cells (Fig. 5D, Fig. S3D).

**Fig. 6:**
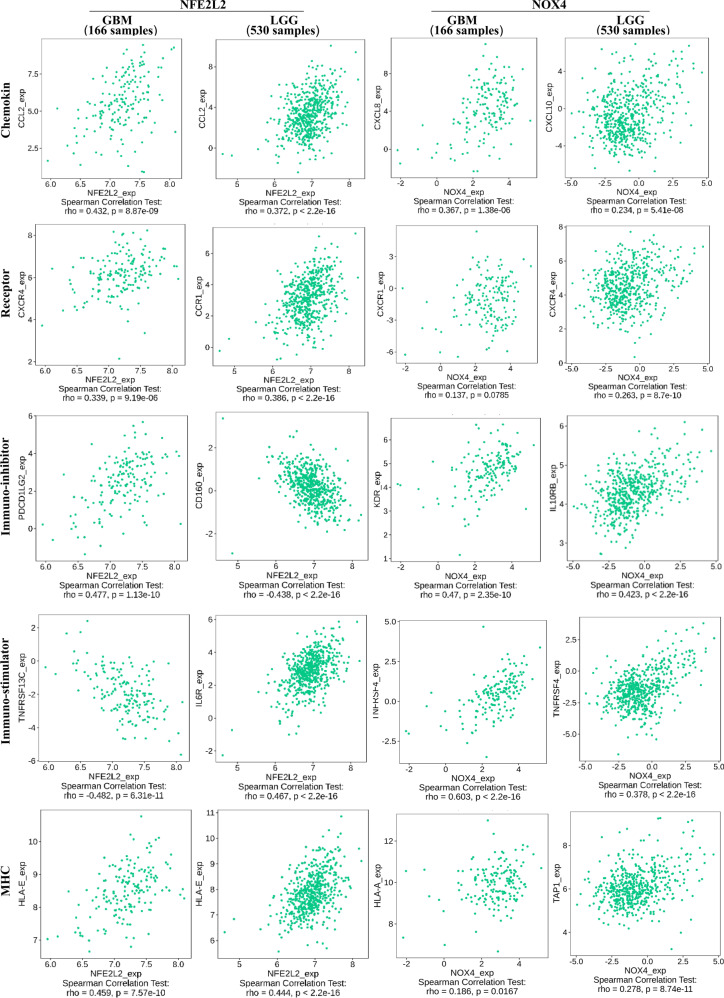
Spearman correlation of between NFE2L2 or NOX4 expression and immunity including chemokines, receptor, immune-stimulator, immune-inhibitor and MHC in glioma (GBM and LGG)

To analyze the association of the two genes and immunogenic features more fully, we validated the correlation of *NFE2L2* and *NOX4* expression with cytokines, MHC, receptor, immune-inhibitor and immune stimulator in GBM and LGG, respectively. As shown in Fig. 6 and Fig. S4, among the 166 GBM samples and 166 LGG samples analyzed for cytokine and MHC expression, the expression of CCL2 and HLA-E was most significantly positively correlated with the *NFE2L2*. For receptors, immuno-inhibitors, and immuno-stimulators, the expression of immune factors is different in GBM and LGG. In GBM, CXCR4 and PSCD1LG2 were positively correlated with *NFE2L2*, and TNFRSF13C was most significantly negatively correlated. While in LGG, CCR1 and IL-6R were positively correlated with *NFE2L2*, CD160 was negatively correlated. As for the *NOX4*, the results showed that TNFRSF4 expression of immuno-stimulators both in GBM and LGG was most significantly positively correlated with gene expression. However, in GBM, the chemokines of CXCL8, a receptor of CXCR1, an immuno-inhibitor of KDR, and MHC of HLA-A were positively correlated with *NOX4*. While in LGG, the chemokines of CXCL10, the receptor of CXCR4, an immuno-inhibitor of IL-10RB, and MHC of TAP1 were most significantly positively correlated with *NOX4*.

## Discussion

Ferroptosis is a newly found programmed cell death that relies on iron ion that can promote lipid peroxidation, which is different with apoptosis or necrosis [17]. The former researches have explored that it is common that high levels of iron are related in a variety of cancer cells rather than in normal cells [49–53]. Moreover, cell death, especially ferroptosis, is critical for glioma development, and it affects pathological classification and sensitivity to chemoradiotherapy. Therefore, cell death is a very promising anticancer strategy to overcome treatment resistance [23–25]. Temozolomide is the most widely used chemical in glioma treatment and has been reported to kill tumor cells through various pathways, including ferroptosis [15, 54, 55]. According to the above evidence, ferroptosis-related drugs with temozolomide could prolong PFS and even OS in the treatment of glioma. As such, ferroptosis is a potential novel approach for glioma treatment. Therefore, it is expressive to analyze the association of ferroptosis-related genes in glioma patients and their prognostic value.

In this study, we first identified 5 ferroptosis-related genes that are closely related to glioma based on the Ferrbe database. Then, we explored the prognostic significance of the 5 ferroptosis-associated genes in glioma by analyzing the gene expression and survival curve of glioma patients. Our results verify that *KEAP1* and *NFE2L2* display clearly higher expression in glioma (GBM and LGG) tissues than in adjacent normal tissues. And the *NOX4* showed a nearly 20-fold increase only in GBM. Furthermore, disease-free survival (DFS) and overall survival (OS) were performed by GEPIA (Fig. 2A). Notably, the DFS and OS were significantly shorter in patients with high expression of *NFE2L2* and *NOX4* than in those with low expression. However, *KEAP1* expression was not significantly associated with the survival curve.

In 2016 WHO categorization, codeletion of chromosomal arms 1p/19q (1p/19q codeletion) and isocitrate dehydrogenase 1 or 2 (*IDH1* or *IDH2*) were included in the diagnostic typing for glioma classification [2, 56]. Recurrently mutated genes *IDH1*, *IDH2,* and 1p/19q are prominent identified factors in prognosis of LGGs in clinical application [11–13]. Our analysis proved that low *NFE2L2* and *NOX4* expression in glioma patients from the TCGA database were significantly related to better pathologic stage and histological grade (Fig. 3). More importantly, we observed that *NFE2L2* and *NOX4* expression have a prognostic value in glioma with AUCs of 0.929 and 0.813, respectively. All results revealed that *NFE2L2* and *NOX4* appeared to be risk factors in glioma. This indicates that the *NFE2L2* and *NOX4* expression-based signatures can better predict the prognosis of glioma.

Tumor microenvironment (TME), the place for tumor birth and growth, play a meaningful role in cancer diagnosis and determining therapeutic options. Moreover, lots of current researches have intimated that the curative effect of immunotherapy is related to the immunogenic TME [32, 57]. Thus, a recondite understanding of the TME in glioma could contribute to digging new ominous markers. We further analyzed the correlation between expression of *NFE2L2* and *NOX4* and immunogenic features, respectively. We found that *NFE2L2* was positively correlated with macrophages and nTreg cells and destructively interrelated with B cells and CD8 naïve cells in GBM. Similarly, *NFE2L2* expression was specifically related to macrophage and Th2 cells and negatively correlated with CD8 naïve and NKT cells in LGG. While *NOX4* expression levels were significantly positively associated with macrophage T cells and negatively correlated with CD4 T cells in GBM. However, in LGG, the high expression levels of *NOX4* could significantly increase gamma delta T cells, Th1, and cytotoxic T cells. Although macrophages have the potential to kill tumors, tumor cells can promote tumor cell expansion and growth by binding to SIRPa on the surface of macrophages in tumor-infiltrating areas through high expression of CD47 [58]. Besides, nTreg and Th2 cells play immunosuppressive functions in TME. However, immune cells such as CD8, NKT, and CD4 play important antitumor immune effects in the TME. These observations suggest that the glioma microenvironment with high expression of *NFE2L2* or *NOX4* was in an immunosuppressive state that confirmed immune system is meaningful to glioma development.

In addition, we validated the correlation of *NFE2L2* and *NOX4* expression with immune cytokines, MHC receptors, immune inhibitors, and activators in GBM and LGG to acquire an extensive understanding of the association between gens and immunogenicity signatures. Interestingly, in GBM, *NFE2L2* expression was positively correlated with many MHCs, cytokines, and other immune molecules such as CCL2, HLA-E, and CXCR4. While in LGG, more immune molecules were positively associated with *NFE2L2* expression, such as CCL2, CCR1, and IL-6R. Similarly, in GBM and LGG, *NOX4* expression was positively correlated with more than 50 immune molecules, such as TNFRSF4, CXCL8, HLA-A, and IL-10RB. Collectively, all results further strongly indicate that *NFE2L2* and *NOX4* play important roles in tumor immunity.

In summary, we systematically investigated the expression patterns of ferroptotic gene in glioma and the relationship to patient outcome. The results of current research point that the overexpression of *NFE2L2* and *NOX4* correlates with poor prognosis in clinical treatment, particularly in LGG. In addition, the high expression of *NFE2L2* or *NOX4* was in an immunosuppressive state, and it correlated with tumor immunity in glioma. Therefore, *NFE2L2* and *NOX4* may function as a risk prognosis biomarker for glioma and play a vital role in immune infiltration and tumor immunity.

## Supplementary information

supportment information

Figure S1

Figure S2

Figure S3

Figure S4
