# Supplementary material for: Ferroptosis-related NFE2L2 and NOX4 Genes are Potential Risk Prognostic Biomarkers and Correlated with Immunogenic Features in Glioma
Source: Cell Biochem Biophys. 2023 Jan 11;81(1):7–17. doi: 10.1007/s12013-022-01124-x (PMC9925512; doi:10.1007/s12013-022-01124-x)
Supplement: Supplementary file 1 — supportment information [file 12013_2022_1124_MOESM1_ESM.docx]

**Ferroptosis-related NFE2L2 and NOX4 genes are potential risk prognostic biomarkers and correlated with immunogenic features in glioma**

Li Lin^#^, Xiaona Li^#^, Shunda Zhu^#^, Qingshan Long, Yongzhen Hu, Liyang Zhang, Zexin Liu, Bo Li, Xuesong Li*

Huizhou Third people’s hospital, Guangzhou medical university, Huizhou, 516002, Guangdong, P.R. China

Corresponding author: lxs75cedar@163.com


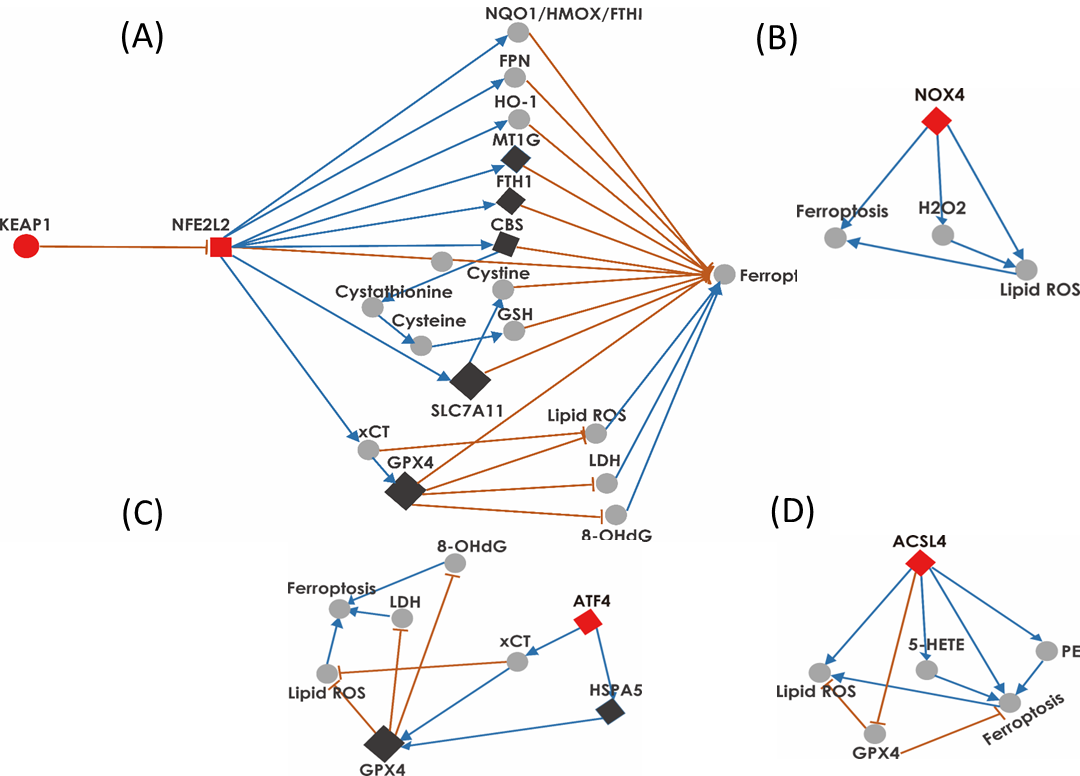


Fig S1 Ferroptosis-associated genes （marked in red）participate in the ferroptosis signaling pathway.


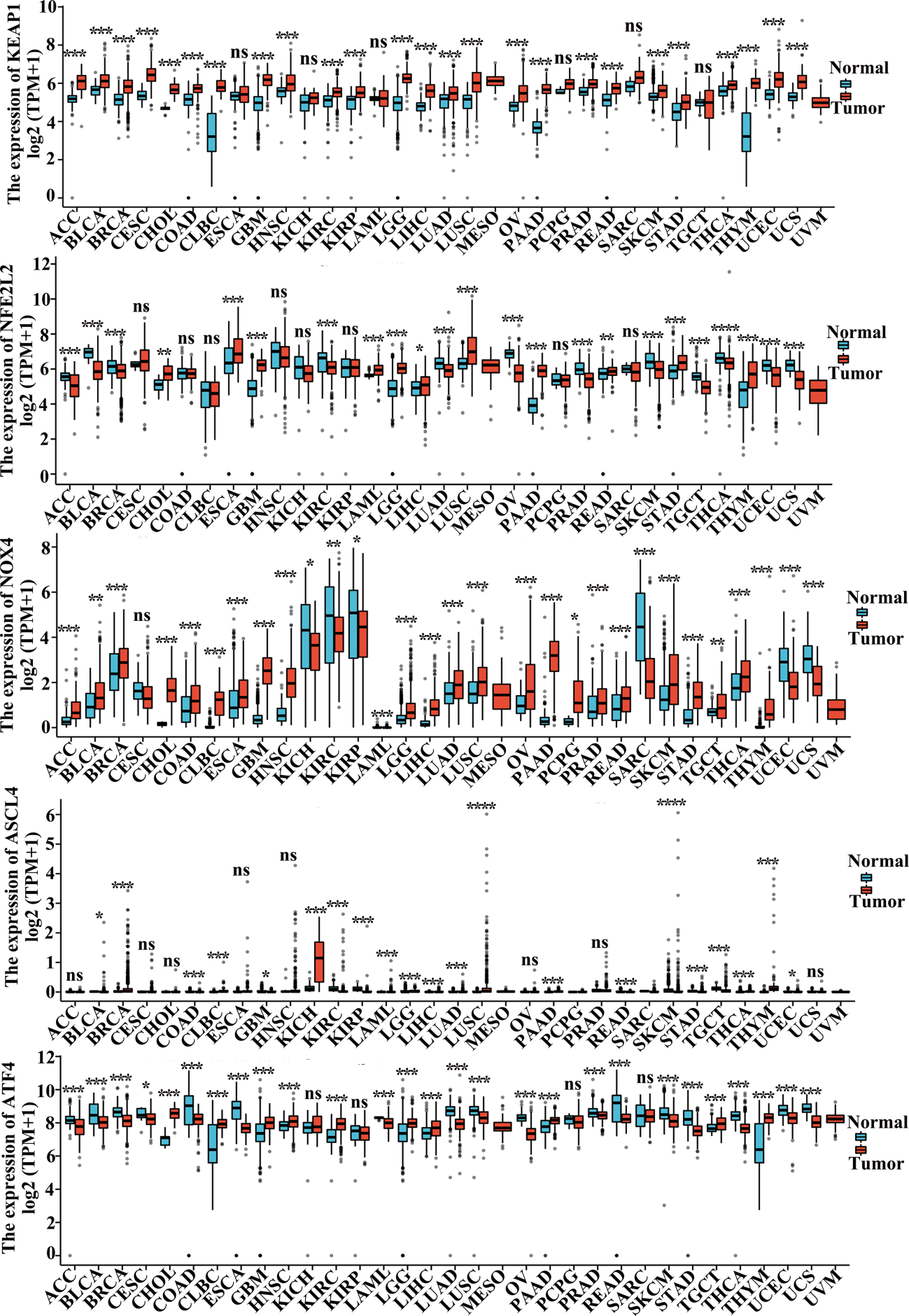


Fig S2 Box plot show ferroptosis-associated 5 genes mRNA expression in tumor (red) and normal (blue) tissue sample corresponding to 33 cancer types from TCGA database. (*p<0.05; **p<0.01; ***p<0.001)


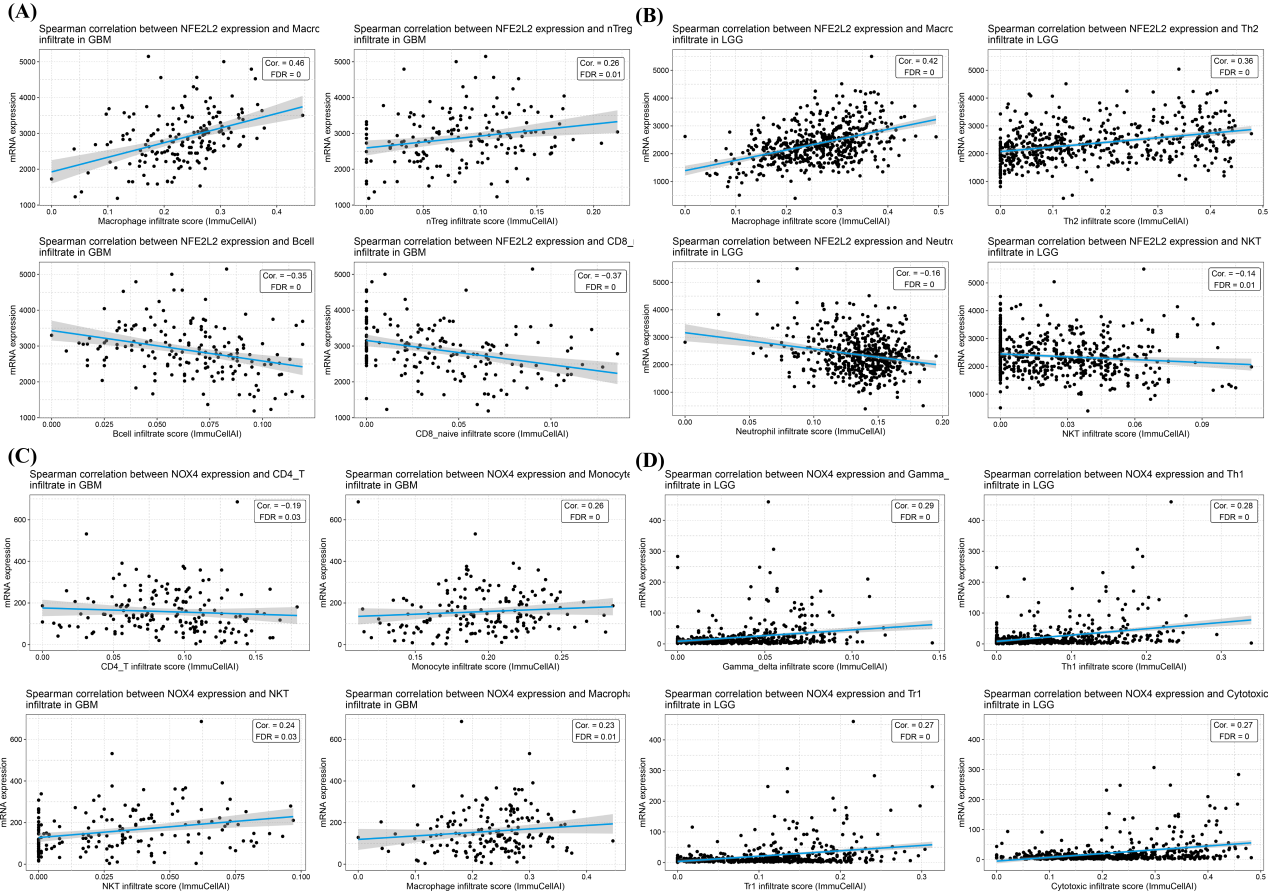


Fig S3 Spearman correlation between NFE2L2 expression and significantly infiltrated immune cells in GBM (A) and LGG (B) ; Spearman correlation between NOX4 expression and significantly infiltrated immune cells in GBM (C) and LGG (D).


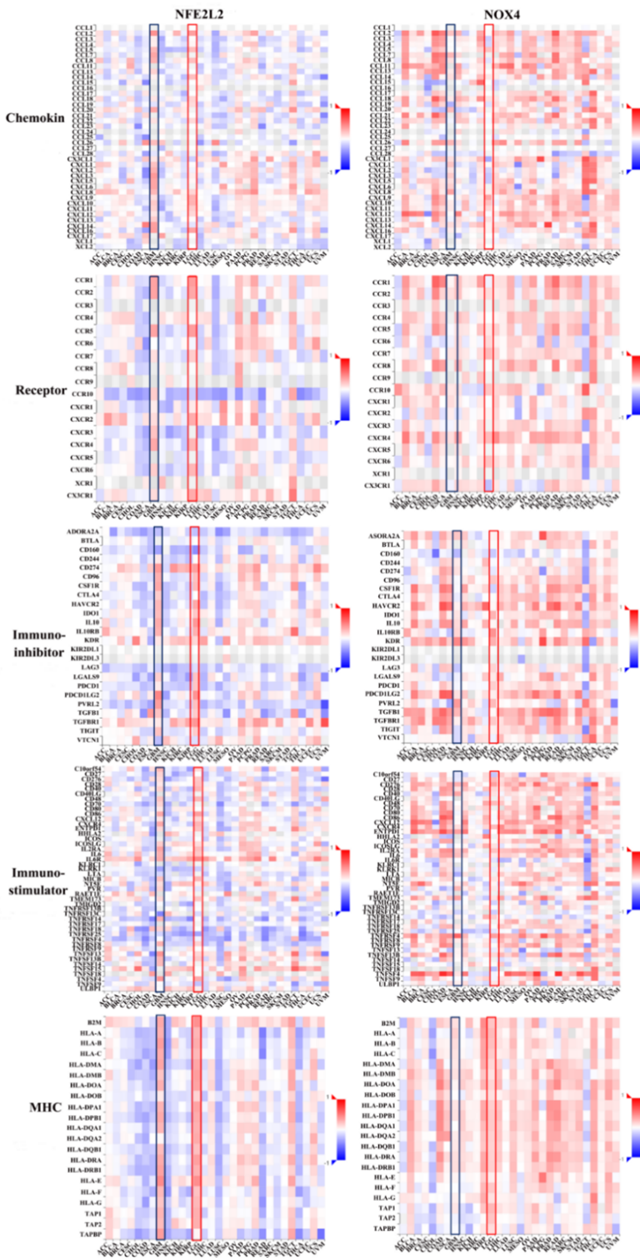


Fig S4 The heatmap of correlation analysis between *NFE2L2* or *NOX4* expression and immunity including chemokines, receptors, immune-stimulators, immune-inhibitor, and MHC in glioma (GBM and LGG). Red represents positive correlation, blue represents negative correlation.
