# Supplementary figures and images for: Ferroptosis-related NFE2L2 and NOX4 Genes are Potential Risk Prognostic Biomarkers and Correlated with Immunogenic Features in Glioma
Source: Cell Biochem Biophys. 2023 Jan 11;81(1):7–17. doi: 10.1007/s12013-022-01124-x (PMC9925512; doi:10.1007/s12013-022-01124-x)

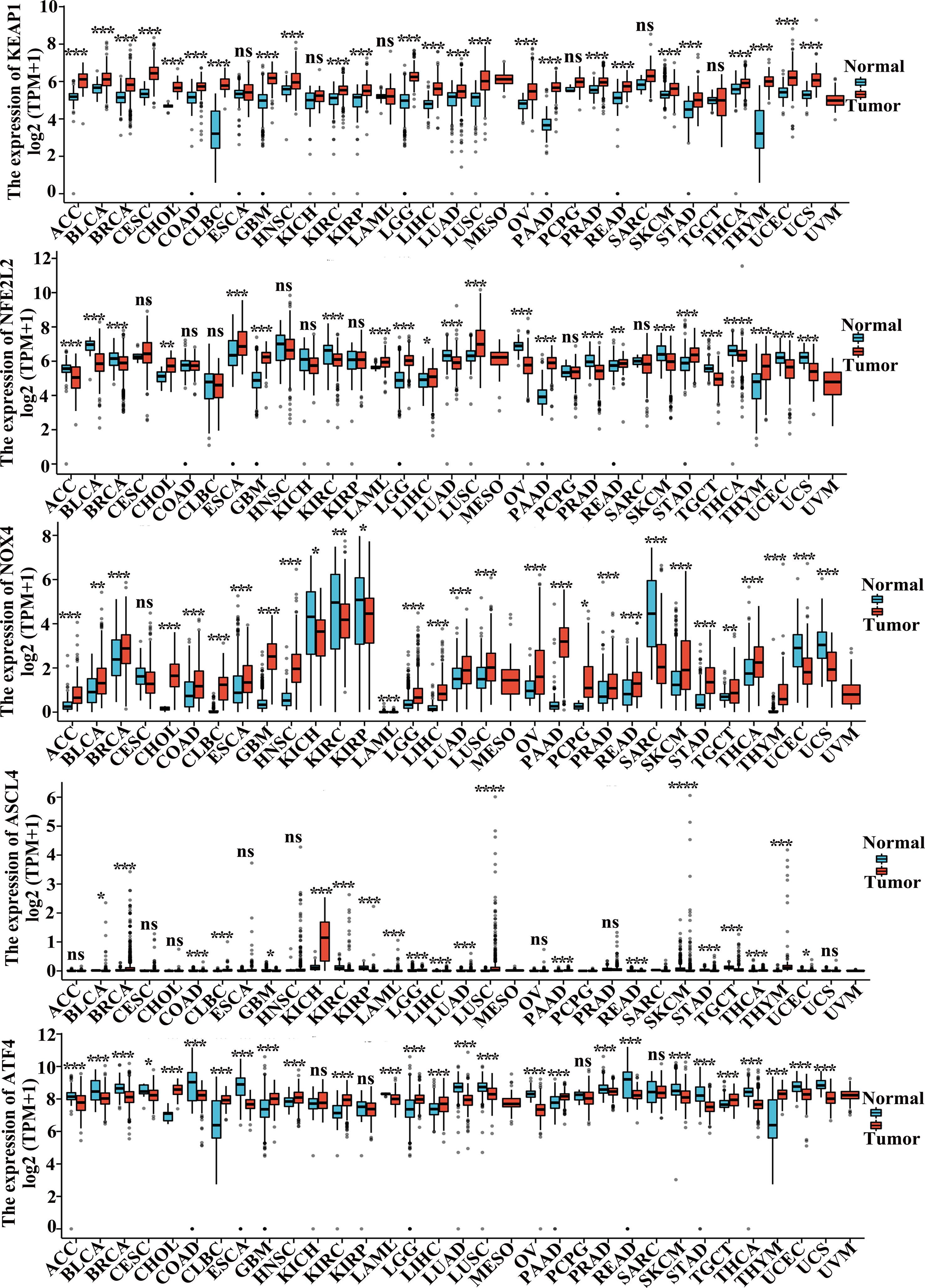

Supplement: Supplementary file 3 — Figure S2 [file 12013_2022_1124_MOESM3_ESM.tif]

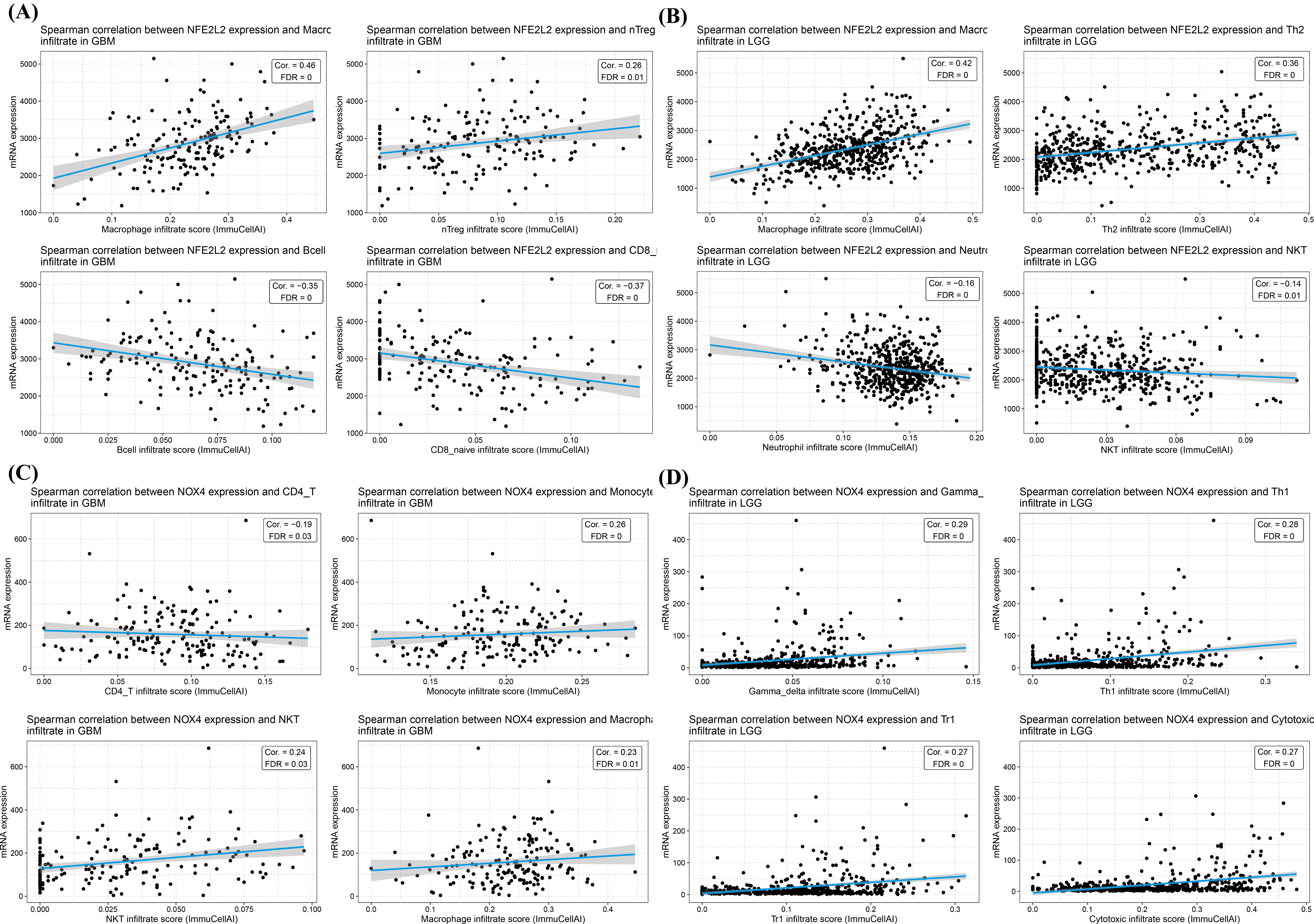

Supplement: Supplementary file 4 — Figure S3 [file 12013_2022_1124_MOESM4_ESM.tif]
